# Broad-spectrum lytic potential of endolysins derived from prophages of clinical *Salmonella* isolates

**DOI:** 10.3389/fmicb.2026.1922952

**Published:** 2026-08-14

**Authors:** Yuhui Liu, Xiuxiu Zeng, Hui Su, Jianing Zhao, Jiayi Dong, Fanfan Yang, Shuo Ye, Wenping Lin, Yuanbin Yang, Yuechao Sun

**Affiliations:** 1 Ningbo Center for Disease Control and Prevention, Ningbo, Zhejiang, China; 2 Ningbo No. 2 Hospital, Wenzhou Medical University, Ningbo, Zhejiang, China; 3 College of Biological and Environmental Sciences, Zhejiang Wanli University, Ningbo, Zhejiang, China; 4 Shandong New-line Biotech Co. Ltd., Weifang, Shandong, China

**Keywords:** antimicrobial resistance genes, bactericidal activity, biocontrol agents, endolysins, phylogenetic analysis, prophages, *salmonella*, virulence factors genes

## Abstract

In the post-antibiotic era, alternative therapeutic strategies were urgently needed. Here, we characterized 514 prophages from 105 clinical *Salmonella* isolates, assessing their carriage of antimicrobial resistance (AMR) genes and virulence factors (VFs) to evaluate biosafety and evolutionary dynamics. Our analyses revealed significant correlations between the abundance of each prophage and host serotypes, as well as between prophage-borne AMR genes and host age or serotype background. Phylogenetic analysis showed that most prophages are related to known *Salmonella* phages, though a subset shares homology with *Burkholderia* viruses, suggesting inter-generic evolutionary connections. A comprehensive screening of prophage-encoded proteins identified a large repertoire of endolysins, with 80.93% of prophages carrying at least one such enzyme. Sequence-based clustering grouped these endolysins into seven families, three of which are widely distributed across the isolate collection. Structural modeling indicated that representative enzymes from these major groups are structurally analogous to thermostable, broad-spectrum lysozymes, which revealed the potential of the proteins as alternatives to antibiotics for treatment. To validate the therapeutic potential of prophage-derived lytic enzymes, we expressed the candidate endolysin *Lys2823* and demonstrated its lytic activity against outer-membrane-permeabilized *Salmonella*. These results indicate that *Lys2823* holds promise as a biocontrol agent for the prevention and treatment of *Salmonella* infections, thereby contributing to food safety and public health. This study provides experimental evidence supporting the development of prophage-derived endolysins as novel antimicrobial agents.

## Introduction

*Salmonella* is a Gram-negative bacterium that causes typhoid fever and enteritis (Wang et al., 2025). In Korea, 6,449 cases of salmonellosis were reported from 2019 to 2023 (Oh et al., 2025). In the past 30 years, antimicrobial resistance in *Salmonella* has exhibited a significant upward trend (Wang et al., 2025). In Guizhou, China, 732 multidrug-resistant *Salmonella* isolates *were* reported between 2019 and 2023 (Wu et al., 2025). In the WHO Bacterial Priority Pathogens List, fluoroquinolone-resistant *Salmonella* is designated as a high-priority pathogen (World Health Organization, 2024). In the postantibiotic era, high investments in antibiotic development have only yielded low-efficacy drugs, highlighting the need for novel therapies (Gordillo Altamirano and Barr, 2019).

The growing drug-resistant *Salmonella* crisis has revitalized research into alternatives, with one of the most promising avenues being phage therapy (Torres-Boncompte et al., 2025). Currently, multiple strains of virulent phages have been isolated and are capable of lysing multidrug-resistant (MDR) *Salmonella* (Torres-Boncompte et al., 2025; Fang et al., 2024). Five isolated phages in one study lysed 12 different *Salmonella* serovars, including Enteritidis and Typhimurium (Torres-Boncompte et al., 2025). In chicken models, phage therapies restrained *Salmonella* in the gastrointestinal tract in 12 published studies (Zhou, K. et al., 2023). In comparison to phage research, the library of prophages in *Salmonella* remains unexplored. Most medical research on prophages in *Salmonella* has characterized prophages and identified antimicrobial resistance (AMR) and virulence factors (VFs; Yates et al., 2024; Ksibi et al., 2022; Trofeit et al., 2023; Fong et al., 2022). One study investigated the activation of prophages to verify phage therapy security (Trofeit et al., 2023). The potential of prophages for therapeutic applications needs further investigation.

The clinical applications of prophages include their use as scaffolds for therapeutic phage engineering in synthetic biology and as a source of lysozymes (Adamczyk-Popławska et al., 2023; Meile et al., 2022; Monteiro et al., 2019; Zhou, S. et al., 2023). In addition, prophages can be induced to transition from lysogeny to lysis in response to environmental changes or appropriate triggering signals (Bruce et al., 2021). In a previous study, Bacteriophage Recombineering of Electroporated DNA (BRED) used prophages as scaffolds and successfully treated a patient with drug-resistant *Mycobacterium abscessus* with the engineered phages (Dedrick et al., 2019). Some prophages can integrate into the host chromosome and restrain bacterial virulence; for example, prophages MP22 and D3112 can reduce the pathogenicity of *P. aeruginosa* lysogens (Vander Elst et al., 2020; Monteiro et al., 2019). Prophage-derived endolysins can cause severe cell lysis in bacterial hosts, which can serve as a replacement strategy for traditional antibiotics (Zhou, S. et al., 2023).

In our research, we verified the viability of *Salmonella* prophages as a novel therapy by analyzing their safety and effectiveness. The genomes, AMR profiles, VFs, and evolutionary relationships were characterized to explore the application of these prophages. We also identified endolysins from the prophage library as alternatives to antibiotics and verified their potential lytic properties.

## Materials and methods

### Data collection

After patients were diagnosed as *Salmonella* carriers, fecal and blood samples were collected. After the *Salmonella* were cultured and purified, sequencing and assembly were performed. The collection process spanned from 2022 to 2024, resulting in a total of 105 usable samples. The collection season for each sample and the ages of the patients were recorded for analysis.

### Host classification by serotypes

We used SISTR (v1.1.1) to identify the serotypes of *Salmonella*^1^ (Yoshida et al., 2016). The phylogenetic analysis by KSNP and iqtree was based on SNPs (Zhou et al., 2024). The results were visualized on the iTOL online platform.^2^ Based on the phylogenetic tree results and serotypes, the samples were categorized into five groups: Typhimurium type (TYP), Goldcoast&Rissen type (G&R), Enteritidis type (ENT), London type (LON), and Unassigned.

### Prophage isolation and statistical analysis

The PHASTEST docker image detected the prophages, and we removed the incomplete prophages (Wishart et al., 2023). Only intact (PHASTEST score > 90) and questionable prophages (PHASTEST score = 70–90) were collected and included in the subsequent analysis. The quantity of prophages in each sample, along with the season of sample collection, patient age, and bacterial serotypes, was analyzed by the Wilcoxon test and Bonferroni correction to assess whether significant differences existed between the different groups (*p* < 0.05) using Python. *W*-values are the sum of the ranks in the two groups in the Wilcoxon test. The sample sizes of the sampling season groups were spring (*n* = 20), summer (*n* = 55), fall (*n* = 28), winter (*n* = 1), and unknown (*n* = 1). The sample sizes of the patient age groups were children (*n* = 47), youth (*n* = 12), middle-aged (*n* = 22), elderly (*n* = 20), and unknown (*n* = 4). The sample sizes of the serotype groups were TYP (*n* = 50), G&R (*n* = 29), ENT (*n* = 17), LON (*n* = 7), and unassigned (*n* = 2).

### Antimicrobial resistance (AMR) and virulent factors (VFs) in prophage identification and analysis

AMR and VFs were detected using MMseqs2, then deduplicated and classified in Python (Steinegger and Söding, 2017). The AMR database used was CARD (Comprehensive Antibiotic Resistance Database;^3^ Alcock et al., 2022). The database used for virulence factor identification was VFDB (database of virulence factors;^4^ Liu et al., 2021). The classification of antimicrobial resistance (AMR) was based on gene types. Virulence factors (VFs) were classified according to the role of virulence genes in bacterial infection. The number of AMR and VFs on prophages in each sample, along with the season of sample collection, patient age, and bacterial serotypes, was analyzed using the Wilcoxon test and Bonferroni correction to assess whether significant differences existed between different groups (*p* < 0.05). *W*-values are the sum of the ranks in the two groups in the Wilcoxon test. The sample sizes of the sampling season groups were spring (*n* = 82), summer (*n* = 286), fall (*n* = 141), winter (*n* = 4), and unknown (*n* = 1). The sample sizes of the patient age groups were children (*n* = 262), youth (*n* = 50), middle-aged (*n* = 92), elderly (*n* = 98), and unknown (*n* = 12). The sample sizes of the serotype groups were TYP (*n* = 304), G&R (*n* = 130), ENT (*n* = 44), LON (*n* = 28), and Unassigned (*n* = 8).

### Cluster classification and reference sequences selection

We used vConTACT2 to classify the taxa of prophages and selected suitable reference sequences, including 31 *Salmonella* prophages, 2 *Burkholderia* viruses, and 1 *Rhizobium* phage (Bin Jang et al., 2019). Reference sequence information was collected from NCBI.^5^

### Evolutionary analysis and comparative genomics analysis

To remove duplicates, all prophage sequences were clustered using CD-HIT (v4.8.1) with 99% similarity, and the representative sequences were analyzed (Fu et al., 2012). The whole phylogenetic tree was constructed by MAFFT version 7 (Katoh and Standley, 2013). Based on the tree, the sub-tree was reconstructed using the VICTOR web service (Meier-Kolthoff and Göker, 2017),^6^ and each group in the sub-tree included one or more reference sequences. The groups of prophages and reference sequences in sub-trees were subjected to comparative genomics analysis and visualization using Clinker (v0.0.31; Gilchrist and Chooi, 2021). The functions of orthologous genes in comparative genomics analysis were annotated using eggNOG Mapper (v5.0.2; Huerta-Cepas et al., 2018) and InterProScan (v5.59; Blum et al., 2024).

### Annotation of prophage proteins and endolysins filtered

We used Prokka (1.14.6) to annotate the prophages primarily (Seemann, 2014) and used eggNOG Mapper (v5.0.2; Huerta-Cepas et al., 2018) and InterProScan (v5.59; Blum et al., 2024) for detailed annotation. Based on the standard of PhaLP (Criel et al., 2021), we selected the endolysins from the annotation results and prepared the sequences for further analysis.

### Endolysins cluster by similarity

We used the Enzyme Similarity Tool (EFI-EST)^7^ to build sequence similarity networks (SSN) to cluster the endolysins in the prophages (Oberg et al., 2023). The E-value threshold was 1E-05, and other settings were kept default. We selected a 100% identity repnode network and colored it using the SSN service on EFI–EST (Oberg et al., 2023). The clusters were visualized using Cytoscape (v3.10.2; Franz et al., 2023).

### Selection of representative sequences and searching for similar proteins via 3D structure

We used CD-HIT (v4.8.1) to select representative sequences in clusters of the SSN result (Fu et al., 2012). AlphaFold2 on the Colab platform^8^ predicted the 3D structures of the representative sequences (Mirdita et al., 2022), and PDB search on the Dali server^9^ was conducted to identify proteins with the closest structural similarity (Holm et al., 2023). The structures of proteins were visualized using UCSF ChimeraX (v1.8).

### Growth conditions, cloning, and purification of *Lys2823*

The *Lys2823* gene from *Salmonella Typhimurium* (SM25) was amplified by PCR. The amplified product was digested with *BamHI* and *XhoI*, ligated into the pGEX-6P-2 expression vector (Miaoling, China), and transformed into *E. coli* BL21 (DE3) pLysS by heat shock (42 °C for 30 s).

Positive transformants were selected on LB agar containing 100 μg/mL ampicillin and 34 μg/mL chloramphenicol, followed by expanded culture. Protein expression was induced with 0.5 mM IPTG, followed by incubation at 28 °C and 160 rpm for 24 h. Cells were harvested, lysed, and the target protein was purified using BeyoGold™ GST Glutathione spin columns, followed by verification of the purified *Lys2823* protein via SDS-PAGE, aliquoting, and storage at −80 °C (Taj et al., 2025).

### Antibacterial effects of Endolysin *Lys2823*

The lytic activity and characteristics of *Lys2823* were determined via a turbidity reduction assay (TRA; Lim et al., 2014; Becker et al., 2009). In brief, *Salmonella Typhimurium* (SM25) was cultured to the logarithmic growth phase, followed by the addition of chloroform to a final concentration of 1%, centrifuged, and washed twice with Tris–HCl at pH 8.0. Tris–HCl was added to the treated bacterial precipitates; the suspensions were treated with 50 mmol/L Tris–HCl buffer (pH 8.0) instead of *Lys2823* treatment as a negative control. After mixing the recombinant endolysin protein *Lys2823* with the bacterial solution and incubating at 37 °C (Jiang et al., 2021). The decrease in absorbance at 600 nm was monitored using a multimode microplate reader for 60 min, with OD_600_ readings recorded every 10 min.

## Result

### Quantity of prophages is related to *Salmonella* serotypes

**Figure 1:**
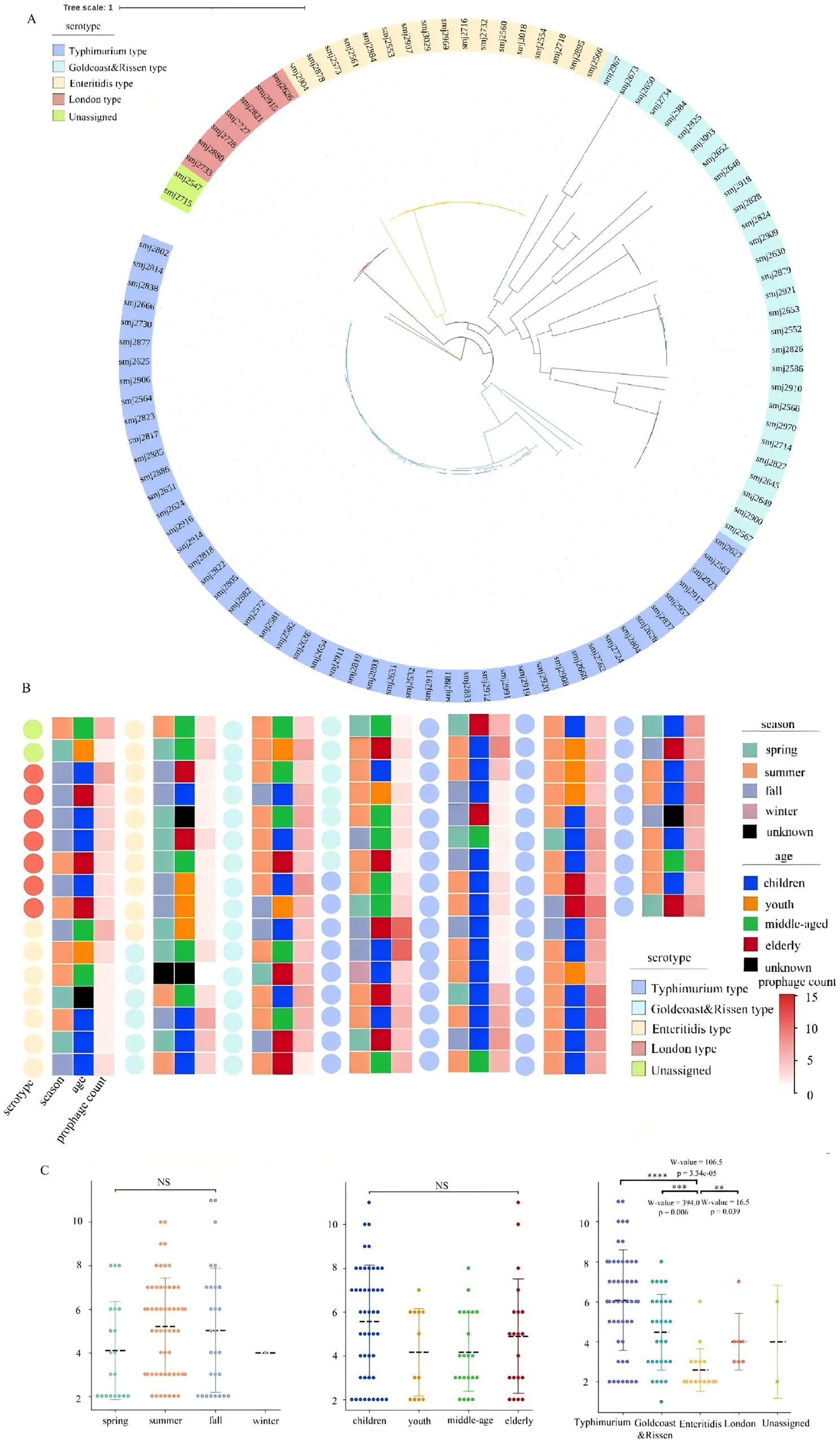
Information on *Salmonella* and analysis of prophage quantity. **(A)** The phylogenetic tree of the strains. Serotypes were classified based on the five main branches. **(B)** Information regarding *Salmonella* and the number of prophages. The first column of dots representing serotypes corresponds to the dots in panel **(A)**, matched in a clockwise manner. The heatmap, arranged from left to right, corresponds to the sampling season, patient age, and quantity of prophages. **(C)** Relationship between the number of prophages and host information. The three scatter plots, arranged from left to right, represent the sampling season, patient age, and serotypes. Each point corresponds to a sample. “NS” indicates no significant difference (corrected *p* > 0.05), while ** indicates a Wilcoxon test and Bonferroni correction result of *p* < 0.05, *** indicates *p* < 0.005, and **** indicates *p* < 0.0005. *W*-values are the sum of the ranks in the two groups. The dashed line represents the mean, and the distance between the colored upper and lower bounds and the dashed line represents the standard deviation.

To study prophages from *Salmonella* genomes, host information, such as sampling time, patient age, serovars, and genomes, was collected for analysis. After sampling and sequencing, we collected 105 strains of *Salmonella* and obtained their whole genomes. Based on the serotype and phylogenetic tree results, the classification revealed that the majority were identified as TYP (*Typhimurium* type; 47.6%) and G&R (Goldcoast & Rissen type; 27.6%). In addition, two strains, which fell outside the main branches, were categorized into the Unassigned group (Figure 1A). Prophages were identified in all strains, with a total of 514 detected. A multivariable heatmap integrated data on serovar classification, sampling season, patient age group, and quantitative prophage carriage profiles across all isolates (Figure 1B). Based on the Wilcoxon test, the quantity of prophages was unrelated to the sampling season or the age of the patients (Figure 1C).

Notably, significant variations in the quantity of prophages existed among different serotypes (Figure 1C). The quantity of prophages in TYP was significantly higher than in other serovars. Overall, the *Typhimurium* prophages were the most common prophages among all *Salmonella* serotypes we collected. The *Salmonella* serotype was therefore associated with the number of prophages.

### AMR and VFs in prophages are associated with host serotype

**Figure 2:**
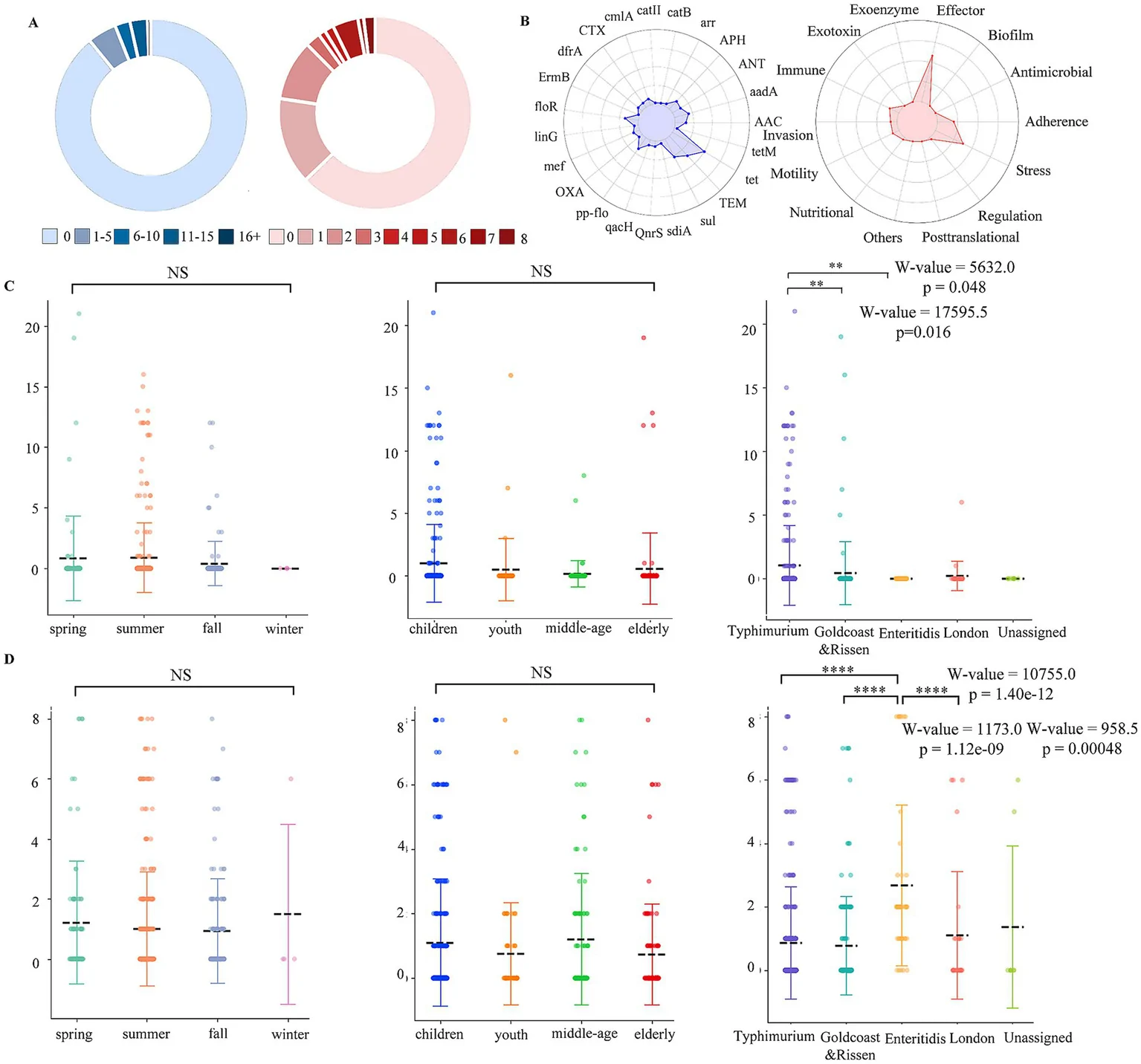
AMR genes and VFs in prophages, and the relationship between their quantity and strain information. **(A)** The proportion of prophages containing varying numbers of *AMR* genes and VFs. The blue pie chart shows *AMR* genes, and the red one shows VFs. **(B)** The types of *AMR* genes and VFs, and the proportion of prophages with the corresponding genes. The innermost circle of the radar chart represents 0%, while the outermost circle indicates 100%, with each layer representing 25%. The blue radar chart shows *AMR* genes, and the red one shows VF. **(C)** The relationship between the number of AMR genes on prophages and strain information. The three scatter plots, arranged from left to right, represent the sampling season, patient age, and serotypes. Each point corresponds to a prophage. “NS” indicates no significant difference (corrected *p* > 0.05), while ** indicates a Wilcoxon test and Bonferroni correction result of *p* < 0.05. *W*-values are the sum of the ranks in the two groups. The dashed line represented the average, and the distance between the colored upper and lower bounds and the dashed line represents the standard deviation. **(D)** The relationship between the number of VFs on prophages and strain information. The three scatter plots, arranged from left to right, represented the sampling season, patient age, and serotypes. Each point corresponded to a prophage. “NS” indicated no significant difference (corrected *p* > 0.05), while **** indicated a Wilcoxon test and Bonferroni correction result of *p* < 0.0005. *W*-values are the sum of the ranks in the two groups. The dashed line represents the average, and the distance between the colored upper and lower bounds and the dashed line is the standard deviation.

To ensure the safety of phage therapy, it is crucial to identify AMR (antimicrobial resistance gene) and VFs (virulence factors) in phages. Among the 514 prophages, 458 were found to lack AMR, 323 were devoid of VF, and 304 lacked both *AMR* and *VF* genes (Figure 2A). AMR included *AAC, aadA, ANT, APH, arr, catB, catII, cmlA, CTX, dfrA, ErmB, floR, linG, mef, OXA, pp-flo, qacH, QnrS, sdiA, sul, TEM, tet, tetM*, and the three with the highest proportions were *tet, TEM,* and *sul* (Figure 2B). The roles of virulence factors (VFs) associated with prophages included adherence, effector delivery systems, immune modulation, invasion, motility, nutritional/metabolic factors, regulation, and stress survival. Among these, the primary functions were effector delivery systems and stress survival (Figure 2B).

The quantity of *AMR* genes carried by prophages was correlated with host serotype. Substantial differences in the quantity of *AMR* genes were observed between the TYP group and the G&R and ENT groups. However, no significant differences in *AMR* gene carriage were observed among the other groups (Figure 2C). Based on the sampling season, patient age, and host serotype groupings, the Wilcoxon rank-sum test and Bonferroni correction revealed that the quantity of VFs in prophages was also correlated with host serotype. Substantial differences in the quantity of VFs were observed between the ENT group and the TYP, G&R, and LON groups. No significant differences in the quantity of VFs were observed across the different sampling seasons and patient age groups (Figure 2D). The relationship between *AMR* genes and serotypes indicated that prophages from the TYP group may have a higher possibility of carrying *AMR* genes. The relationship between VFs and serotypes indicated that prophages from the ENT group may have a higher possibility of carrying VFs.

### Prophages display collinearity with *Salmonella* phages and *Burkholderia* viruses

**Figure 3:**
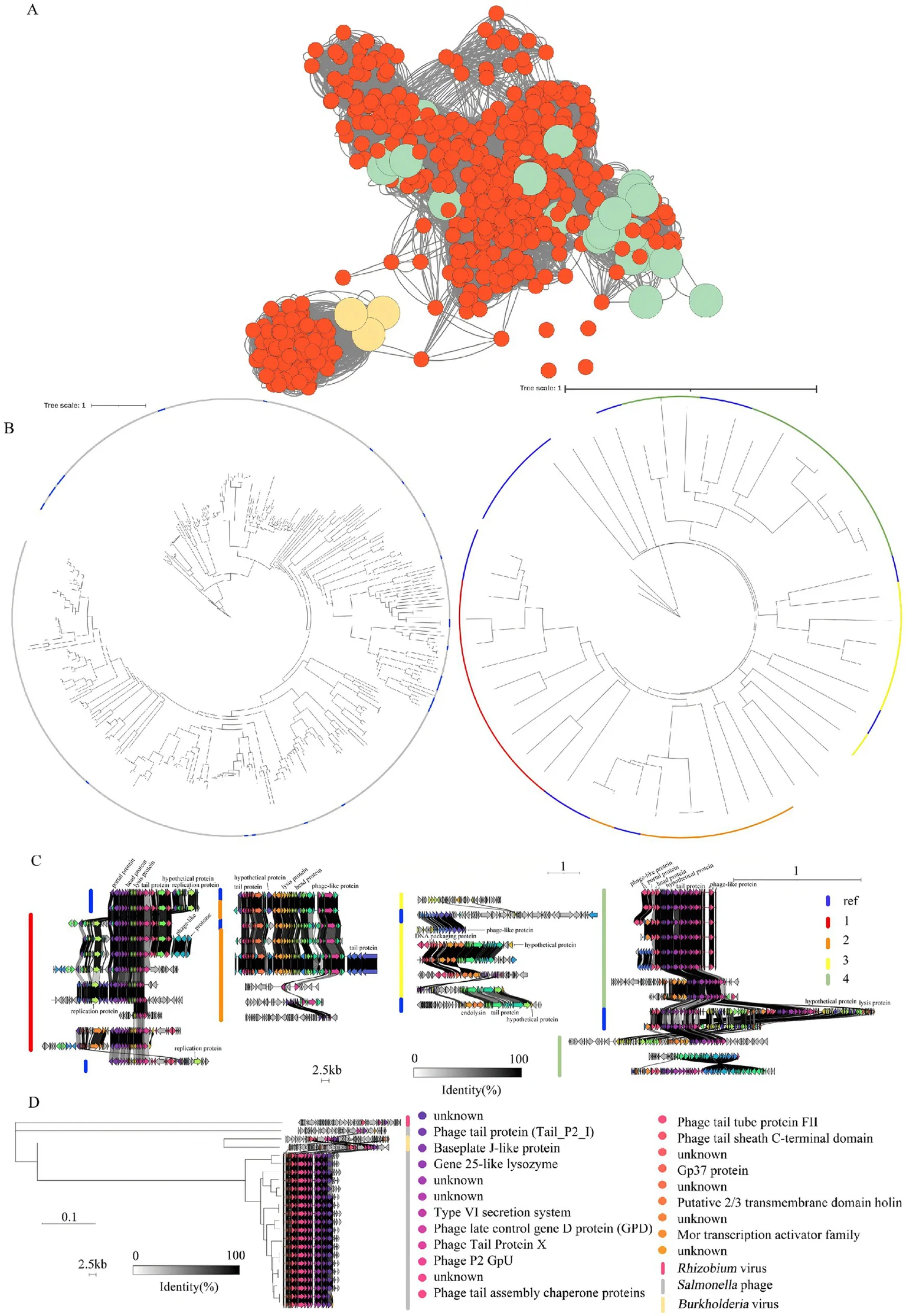
The evolutionary and collinearity relationships of prophages. **(A)** Clusters between prophages with *Salmonella*, *Burkholderia,* and *Rhizobium* viruses. Each red circle represents a single prophage. Other circles represent reference viruses. The size of the circle is positively correlated with the number of prophages related to the viruses. **(B)** Phylogenetic tree of prophages and *Salmonella* viruses. The left tree includes all genomes, while the right tree is the sub-tree from the left one. The color of the scale represents the type of node. For the left one, grey is prophage. For the right one, red is group 1, orange is group 2, yellow is group 3, and green is group 4. The blue node always represents the reference sequence in panel **(B)**. **(C)** Collinearity relationships within the groups. From left to right are groups 1, 2, 3, and 4. The color of the line represents the type of sequence. Orthologous genes were identified by sequence similarity and are color-coded. Identical colors in different groups represent different homologous genes. Grey regions represent other proteins. The functions of orthologous genes are annotated on the figure. To enhance the visual appeal of the figure, the word “protein” in the marks has been omitted. **(D)** The hylogenetic tree and collinearity analysis of prophages with *Burkholderia* and *Rhizobium* viruses. Orthologous genes were identified by sequence similarity and are color-coded. Grey regions are other proteins. The long scale is the tree scale, and the short one is the gene length scale. The color of the line represents the type of sequence: ink is *Rhizobium* virus, grey is prophages, and light orange is *Burkholderia* viruses.

To identify and characterize prophages, we clustered the 514 prophages and analyzed their taxonomic context. Most of the prophage clusters were identified as *Salmonella* phages. However, a subset (*n* = 78) of prophages exhibited similarity to *Burkholderia* and *Rhizobium* viruses (Figure 3A). After removing sequences with 99% similarity using CD-HIT, we analyzed the evolutionary relationships among 320 prophages and 31 reference phages (Figure 3B). Based on the phylogenetic tree, we selected 38 prophages that were closely related to the reference phages and built a sub-tree for further comparative genomics analysis (Figure 3B). The prophages and reference phages were divided into four groups. The collinearity analysis results indicated that the prophage groups exhibited a high degree of homology with the corresponding reference *Salmonella* phages, suggesting the existence of conserved proteins in *Salmonella* phages (Figure 3C).

In addition, we analyzed the evolutionary relationships and collinearity of the subset of prophages with *Burkholderia* and *Rhizobium* viruses. The analysis results indicated that, except for one prophage, the other prophages in the subset exhibited a high degree of homology with two *Burkholderia* viruses. The annotation of orthologous proteins suggested that the majority were phage tail proteins, tail assembly proteins, and endolysins (Figure 3D). The structural proteins in prophages of *Salmonella* and *Burkholderia* showed homology, indicating that the conservation of structural proteins exists across different phages. Because one of the *Burkholderia* phages, BcepMu, is a Mu-like phage (Samanta et al., 2021), the similar lysis structures in these prophages may represent a Mu lysis system.

### Endolysins in prophages exhibit lytic capability

**Figure 4:**
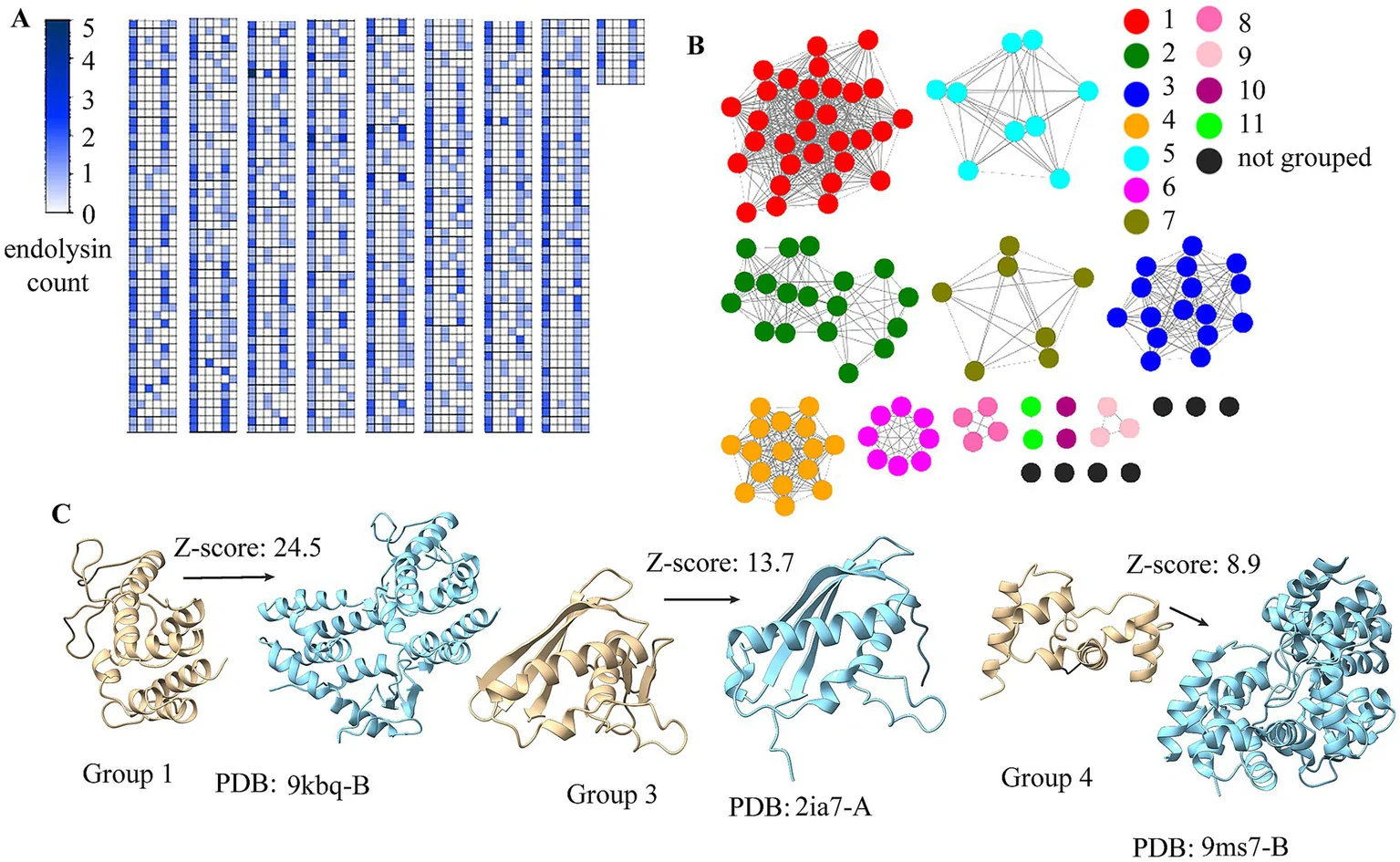
The endolysins in prophages. **(A)** The distribution of endolysins in prophages. Each row represents a prophage containing endolysins, with the columns from left to right indicating the following: Total number of all lysozymes, N-acetylmuramoyl-L-alanine amidase, N-acetylmuramoyl-L-alanine amidase and peptidase, peptidase, N-acetyl-*β*-D-muramidase, and N-acetyl-β-D-muramidase, lytic transglycosylase. The darker the color, the greater the quantity. **(B)** Clusters of endolysin genes according to similarities. Each color corresponds to a specific group of endolysins, while black represents non-clustered genes. The edge linking the two points shows similarities between them. **(C)** The 3D structures of representative proteins and their structurally similar proteins. The yellow proteins are the representative sequences, and blue proteins are the similar reference structures. A higher *Z*-score indicates greater structural similarity between the proteins.

To explore effective endolysins as an alternative to antibiotics, we analyzed the gene library of prophages. The genomes of the prophages were annotated, and we found 619 endolysins in 416 prophages, accounting for 80.93% of all 514 prophages (Figure 4A). The endolysins were categorized into five classes: (i) N-acetylmuramoyl-L-alanine amidase, (ii) N-acetylmuramoyl-L-alanine amidase and peptidase, (iii) peptidase, (iv) N-acetyl-*β*-D-muramidase, and (v) N-acetyl-β-D-muramidase and lytic transglycosylase. Among them, 409 endolysins were classified as N-acetyl-β-D-muramidase, accounting for 66.07% of the total (Figure 4A). According to sequence similarities, the endolysins were clustered, and seven groups contained more than five distinct proteins (Figure 4B). The three protein groups covering the most *Salmonella* were group 1, group 3, and group 4, which existed in prophage regions 85, 79, and 67 *Salmonella*, respectively (Figure 4B). This indicated that the endolysins may be able to lyse a majority of the collected *Salmonella* (*n* = 105), demonstrating broad-spectrum lytic capability of the endolysins, although this remains a predictive inference.

We predicted the 3D structures of the representative sequences of Groups 1, 3, and 4, and identified the most similar proteins in structure. The protein most closely related to the representative sequence of Group 1 was 9kbq-B, a peptidoglycan hydrolase characterized by thermal stability and broad-spectrum activity, which was capable of lysing *Acinetobacter baumannii*. For Group 3, the similar protein was 2ia7-A, a putative tail lysozyme from *Geobacter sulfurreducens.* For Group 4, the protein was 9 ms7-B, a putative phage endolysin from the metagenome of soil (Figure 4C). 9kbq, which is the PDB name of PHAb10, expressed robust lysis activity against *A. baumannii*, *P. aeruginosa*, *E. coli*, *K. pneumoniae*, *Streptococcus suis*, and *E. faecalis* in log-killing assay experiments. Heat treatment at temperatures up to 100 °C for 1 h would increase the lysis ability of PHAb10 slightly (Zhang et al., 2024). These experimental validations demonstrated the broad-spectrum activity and thermal stability of PHAb10, and enzymes with similar structures may have excellent features.

The lytic capability of structurally similar proteins suggested that endolysins may have the capability to lyse bacteria beyond *Salmonella*, exhibiting cross-species lytic activity. In other words, the potential applications of endolysins in prophages extend beyond the lysis of *Salmonella*, as they may also serve as broad-spectrum antimicrobial agents.

### Expression, purification and concentration determination

**Figure 5:**
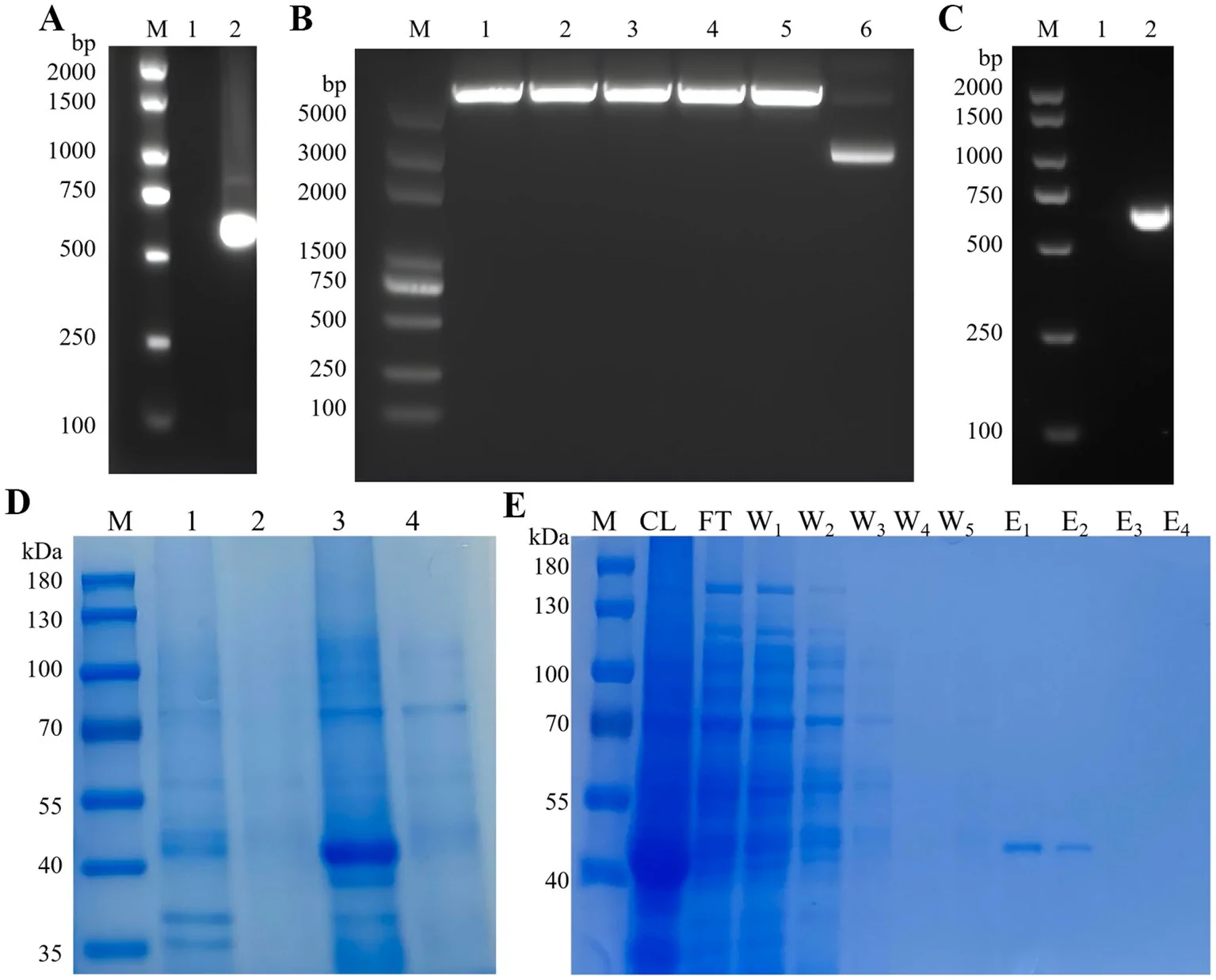
Expression of endolysin *Lys2823*. **(A)** Agarose electrophoresis of PCR-amplified *Lys2823* gene. DL2000 DNA Marker (Lane M), negative Control (Lane 1), PCR-amplified *Lys2823* (Lane 2). **(B)** Results of enzyme digestion of PGEX-6p-2 plasmid. DL5000 DNA Marker (Lane M), digested plasmid (Lane 1–5), negative Control (Lane 6). **(C)** Results of enzyme digestion of recombinant plasmid. DL2000 DNA Marker (Lane M), negative Control (Lane 1), PCR-amplified *Lys2823* (Lane 2). **(D)** Analysis of recombinant endolysin *Lys2823* expression patterns. Standard molecular weight markers (Lane M), the total bacterial lysate without IPTG induction (Lane 1), the supernatant of cell lysates without IPTG induction (Lane 2), the total bacterial lysate with IPTG induction (Lane 3), the cytoplasmic fractions with IPTG induction (Lane 4). **(E)** The purified recombinant endolysin *Lys2823*. Standard molecular weight markers (Lane M), lysate of recombinant strain after induction by IPTG (Lane CL), flow-throughs (Lane FT), wash fractions (Lane W1-W5), eluted fraction of purified *Lys2823* (Lane E1-E5).

PCR amplification of the *Lys2823* gene yielded DNA fragments of the expected size, as shown in Figure 5A. Restriction enzyme digestion of the pGEX-6p-2 plasmid is presented in Figure 5B. The pGEX-6p-2 vector was used to construct a recombinant plasmid containing the target fragment that could be propagated in DH5ɑ. The recombinant plasmid pGEX-6p-2-*Lys2823* was successfully constructed and confirmed by restriction enzyme digestion to yield the expected fragment sizes, as shown in Figure 5C. The recombinant plasmid pGEX-6p-2-*Lys2823* was then transferred into *E. coli* BL21 (DE3) pLysS for the production of the lytic enzyme, and expression of the target protein was induced by the addition of 0.5 mM isopropyl-*β*-D-thiogalactopyranoside (IPTG) at 28 °C. This was followed by further culture for approximately 24 h. Recombinant bacteria without IPTG induction were used as controls. The whole-cell lysate and the soluble supernatant of the lysate of the recombinant strain were collected using ultrasonification and visualized using sodium dodecyl sulfate-polyacrylamide gel electrophoresis (SDS–PAGE). As shown in Figure 5D, this protein was detected in cytoplasmic fractions. The predicted size of *Lys2823* was approximately 42 kDa.

After purification of *Lys2823* with the GST-tag Protein Purification Kit (Beyotime, P2262), we obtained the SDS–PAGE analysis results shown in Figure 5E. The target band and the corresponding positions can be clearly observed. The concentration of *Lys2823* was determined using the BCA Protein Quantification Kit (Vazyme, E112), and the result was approximately 1,191 μg/mL.

### Antibacterial effect of Endolysin *Lys2823*

**Figure 6:**
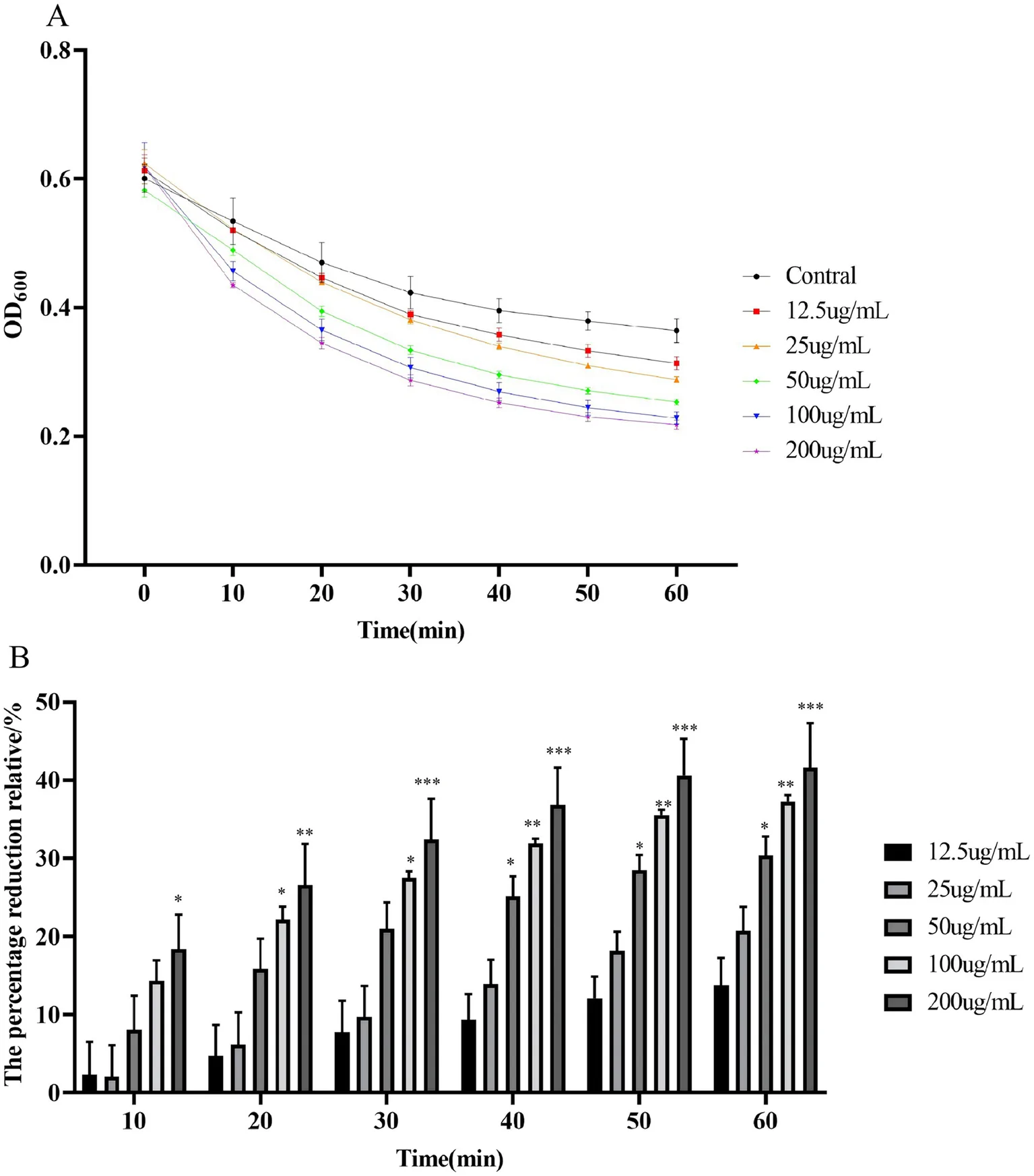
*Lys2823* bacteriolytic activities against *Salmonella Typhimurium* (SM25). **(A)** Lytic activity of *Lys2823* at different concentrations. The concentration of protein used was indicated. Tris–HCl without adding *Lys2823* was used as a control. **(B)** Growth dynamics of *Lys2823* at different concentrations, as monitored by ∆OD_600_ at multiple time points.

After treatment with chloroform (final concentration of 1%), *Lys2823* at a concentration of 12.5–200 μg/mL was added for 60 min. As shown in Figure 6A, the results of the turbidity assay showed that *Lys2823* exhibited antibacterial activity against *Salmonella Typhimurium* (SM25). The results of the turbidity assay showed that *Lys2823* at concentrations of 100 μg/mL and 200 μg/mL could reduce the optical density of *Salmonella Typhimurium* (SM25) by an OD_600_ of 0.2 within 1 h. The turbidity reduction assay demonstrated a significant 40% reduction compared to the control at 50 min with 200 μg/mL *Lys2823* (Figure 6B), indicating the rapid lysis of *Salmonella*.

## Discussion

Prophages are ubiquitous in nature, and nearly half of sequenced bacteria are lysogens. Although prophages do not immediately lyse their hosts, they can attenuate host virulence or serve as scaffolds for phage genome engineering (Monteiro et al., 2019). Endolysins encoded by prophages possess significant potential as enzybiotics due to their high enzymatic activity and abundance (Morais et al., 2022). While Yates et al. (2024) characterized *Salmonella* prophages to assess their distribution and functions, our research analyzed VFs, evolutionary relationships, and endolysins to evaluate prophage safety as well as their ability to infect and lyse bacterial cells. To our knowledge, the application potential of *Salmonella* prophages has not been subjected to such a targeted analysis before this research.

Most prophages exhibited a high degree of homology with the corresponding *Salmonella* phages, which demonstrated the potential of prophages as scaffolds for novel therapies. Homologous recombination, a strategy for phage genome engineering, replaced the tail fiber encoding genes of phage T2 with their homologous counterparts from phage IP008 to broaden the host range (Lv et al., 2023). These prophages have the potential to serve as resources for the homologous recombination technique. Notably, a subset of prophages from *Salmonella* shared homology with *Burkholderia* viruses, and most of the orthologous proteins were structural proteins. *Burkholderia* phage BcepMu and *Salmonella typhi* phage SalMu are Mu-like phages, which all have proteins that control lysogenic or lytic lifestyles, tail genes, and endolysins (Samanta et al., 2021), including the orthologous proteins detected in the prophages. The similarity of structural proteins indicated that a lysis system like the Mu lysis system existed in *Salmonella* prophages, possibly (Chamblee et al., 2022; Toussaint, 2017).

The endolysins exist widely in prophages, and seven groups of enzymes were present in multiple *Salmonella* isolates. According to the analysis of their 3D structure, the representative proteins of the top three groups were similar to lysozymes from other bacteria, such as *Acinetobacter baumannii* and *Geobacter sulfurreducens*. Endolysins exhibit a more extensive bactericidal spectrum than their hosts, as reported in several studies (Jiang et al., 2021; Kim et al., 2022; Deng et al., 2023). Similar enzymes from *Acinetobacter baumannii* were characterized as peptidoglycan hydrolases with thermal stability and broad-spectrum activity without dependence on an outer membrane permeabilizer (Zhang et al., 2024); this structural homology suggests that the identified endolysins may possess comparable thermostability and broad-spectrum properties, warranting further experimental validation. It also suggests that prophages have the potential to lyse hosts via endolysins, demonstrating their effectiveness.

In our research, the endolysins that were present in multiple *Salmonella* strains were selected from prophages and demonstrated their potential as enzybiotics. In the current study, the *Lys2823* endolysin derived from a *Salmonella* prophage was successfully expressed in *E. coli* BL 21 (DE3) pLysS, purified, and identified (Figure 5). *Lys2823* in combination with chloroform exhibited high relative lytic activity against *Salmonella* (Figure 6). However, as this study was based entirely on clinical *Salmonella* strains, the results may be biased toward clinical isolates. While the exclusive use of clinical Salmonella isolates in this study may introduce sampling bias, it also enhances translational relevance, as these strains represent the pathogens most directly responsible for human infections and antibiotic treatment failures. However, this clinical focus underrepresents environmental and zoonotic reservoirs—key sources of emerging serovars and AMR genes—potentially limiting the broad-spectrum applicability of our endolysin library for food safety and veterinary contexts. Nevertheless, we argue that the clinical focus of this study is a justified and strategic choice. The goal of developing endolysins as antimicrobial agents is to address the immediate crisis of multidrug-resistant Salmonella infections in humans. A library derived from clinical isolates provides a targeted and clinically actionable resource for therapeutic design, with a higher likelihood of direct efficacy against circulating pathogenic strains. Future work should complement this approach by expanding the prophage survey to include environmental, foodborne, and veterinary isolates, thereby creating a more comprehensive and resilient endolysin arsenal that can address both clinical and zoonotic threats. In addition, these analytical results were derived from bioinformatics methods, which carry inherent uncertainties associated with in silico simulations. The conservation of structural proteins between *Salmonella* prophages and *Burkholderia* phages suggests the possibility of a lysis system in these prophages. The differences in specific lysis systems across phages require further characterization to investigate the lysis mechanisms for various hosts.

## Data Availability

The datasets presented in this study can be found in online repositories. The names of the repository/repositories and accession number(s) can be found in the article/Supplementary material.

## Footnotes

1
https://github.com/phac-nml/sistr_cmd

2
https://itol.embl.de/

3
https://card.mcmaster.ca

4
http://www.mgc.ac.cn/Vfs/download.htm

5
https://www.ncbi.nlm.nih.gov/

6
https://victor.dsmz.de

7
https://efi.igb.illinois.edu/efi-est/

8
https://colab.research.google.com/github/sokrypton/ColabFold/blob/main/AlphaFold2.ipynb

9
http://ekhidna.biocenter.helsinki.fi/dali/
